# Loss of HOP tumour suppressor expression in head and neck squamous cell carcinoma

**DOI:** 10.1038/sj.bjc.6601952

**Published:** 2004-06-22

**Authors:** F Lemaire, R Millon, D Muller, Y Rabouel, L Bracco, J Abecassis, B Wasylyk

**Affiliations:** 1 Institut de Génétique et de Biologie Moléculaire et Cellulaire, CNRS/INSERM/ULP, 1 Rue Laurent Fries, BP 10142, 67404 Illkirch cedex, France; 2 UPRES EA 34-30, Centre Paul Strauss, 3 rue de la Porte de l'Hôpital, 67085 Strasbourg, France; 3 Exonhit Therapeutics S.A., 65 Boulevard Masséna, Paris F-75013, France

**Keywords:** hypopharynx, homeodomain, development, differentiation

## Abstract

We report that homeodomain-only protein (HOP) is expressed in the suprabasal layer of normal upper aerodigestive tract epithelium and expression strongly decreases in hypopharyngeal carcinoma. Interestingly, HOP has very recently been shown to be a tumour suppressor involved in differentiation, suggesting that HOP may have a similar role in head and neck squamous cell carcinoma (HNSSC).

Head and neck squamous cell carcinoma (HNSCC) occurs through malignant conversion of basal layer epithelial cells of the upper aerodigestive tract (pharynx, hypopharynx and larynx) and the oral cavity. HNSSC is the fifth most common cancer worldwide (Sankaranarayanan *et al*, 1998) and the 5-year survival rate is only 18% for hypopharyngeal carcinoma (Dimery and Hong, 1993). We have performed differential display (DD) (Lemaire *et al*, 2003) and DNA microarray (Cromer *et al*, 2004) analysis of hypopharyngeal tumours to search for new biomarkers and targets for drug design. In this complementary study, we report that expression of homeodomain-only protein (HOP) is strongly decreased in tumours. Interestingly, in a series of recent independent reports, this protein under different names (mOB1/HOP/LAGY/NECC1/SMAP31) has been shown to be involved in development (Adu *et al*, 2002; Chen *et al*, 2002; Shin *et al*, 2002; Asanoma *et al*, 2003; Chen *et al*, 2003) and has been shown to be a potent tumour suppressor (Asanoma *et al*, 2003). We show here that HOP is expressed at a precise stage of epidermoid cell differentiation and its expression is lost in HNSSC, suggesting that HOP may be a general tumour suppressor gene involved in keratinocyte differentiation.

## MATERIALS AND METHODS

Tumour samples, Northerns, Virtual Northerns and real-time quantitative PCR (RT–QPCR) were as described previously (Lemaire *et al*, 2003). Virtual signifies the use in the Northern blots of SMART cDNA synthesised by SMART (Clontech, http://www.bdbiosciences.com/c lontech/). The primer sequences were HOP, 5′-TCAACAAGGTCGACAAGCAC-3′ and 5′- TCTGTGACGGATCTGCACTC-3′; the ubiquitous gene RPLPO, 5′- GAAGGCTGTGGTGCTGATGG-3′ and 5′- CCGGATATGAGGCAGCAGTT -3′. For each PCR, the unknown sample expression level was estimated relative to standard curves using a pool of normal samples. PCR reactions were run at least twice for each sample and results normalised using RPLP0 as an internal control. *In situ* hybridisation (ISH) was performed by a standard technique (Strahle *et al*, 1994; Cau *et al*, 1997). Nonradioactive digoxigenine-labelled RNA probes were synthesised from 15 *μ*g of linearised F1.4-pGEMT-Easy (antisense: Aat2 and SP6 RNA polymerase; sense: Spe1 and T7 polymerase). F1.4-pGEMT-Easy: pGEM-T Easy with a 572 bp insert corresponding to SMAP31 (now HOP transcript variant 1, accession number BC014225, nt 675–1246).

## RESULTS

**Figure 1:**
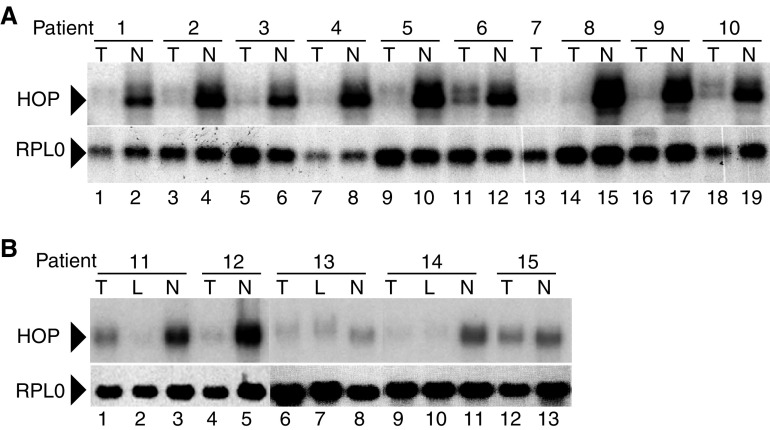
Analysis of HOP RNA expression by Virtual Northern blots (**A**) and Northern blots (**B**). Bars indicate tumour (T) and normal (N) samples from the same patients. Comparisons should be made between matched samples. RPLP0 is the loading control.

We isolated a differentially expressed sequence (F1. 4) in a DD analysis of RNA from HNSCC and matching normal tissue. The sequence corresponded to SMAP31/LAGY, which at that time was annotated in the NCBI database as a clone underexpressed in choriocarcinoma *vs* normal placenta villi (SMAP31) and lung cancer *vs* normal lung (LAGY). More recently, it has been called HOP (Chen *et al*, 2002; Shin *et al*, 2002) since it is a homeodomain-only protein. Homeodomain-only protein was not detected in our previous study (Lemaire *et al*, 2003), apparently because it is not efficiently labelled with the primer mixtures that we used to make focused probes for Reverse Northern analysis. However, in further studies using Virtual Northerns (Northerns with SMART amplified cDNAs), we found a striking decrease in expression in tumours in nine matched tumour-normal pairs (Figure 1A

). The major transcript migrates around 1000 bp, which corresponds to the sizes reported for HOP (1200 bp, (Chen *et al*, 2002; Shin *et al*, 2002)), LAGY (HOP/LAGY transcript variants: 1013 bp, NM_139212; 989 bp, NM_139211, 1265 bp, NM_032495 (Chen *et al*, 2003)), and NECC1/SMAP31-22, SMAP31-R and SMAP31-12 (980 bp, AB059409, 989 bp, AB059410 and 1097 bp, AB059408, respectively, (Asanoma *et al*, 2003)]).

**Figure 2:**
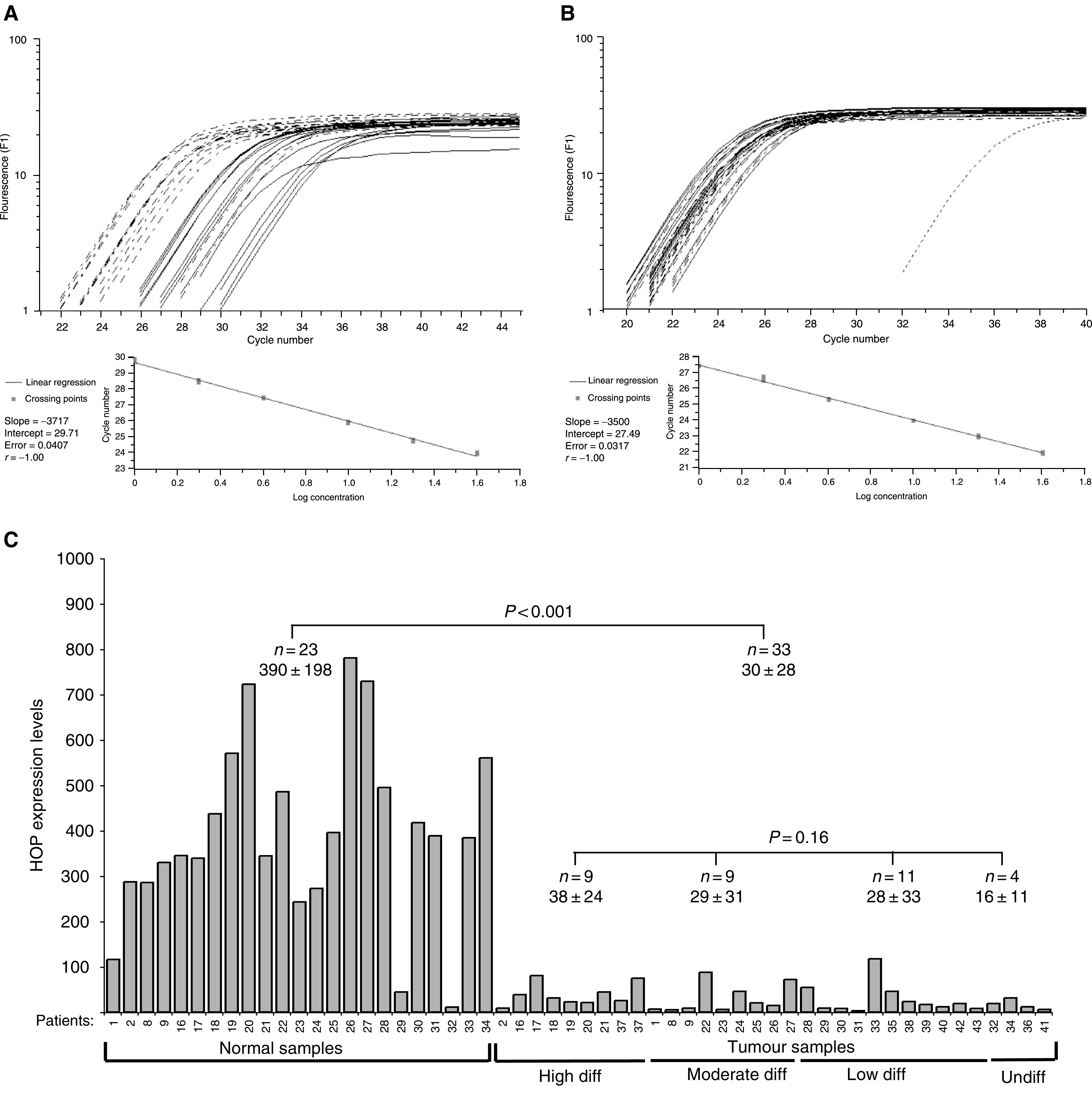
Real-time quantitative RT–PCR. (**A**, **B**) Traces and standard curves of HOP (**A**) and RPLP0 (**B**) analysis for tumour samples (full line) and normal samples (long dotted line). Control without cDNA (short dotted line). (**C**). HOP expression was analysed in 33 hypopharyngeal tumours and 23 matched normal samples by RT–QPCR. HOP expression values were calculated in relative units adjusted to the RPLP0 internal controls. Statistical analysis between tumour and normal samples, and between tumours according to their histopathological differentiation status, were carried out by ANOVA statistical tests.

**Figure 3:**
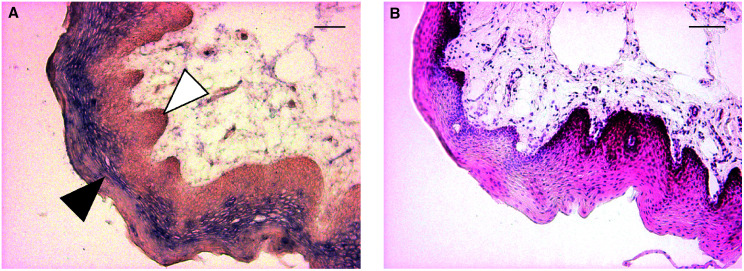
*In situ* hybridisation of normal tissue. The uvula was hybridised with an antisense probe (**A**) or stained with haematoxylin/eosin (**B**). The black arrowhead indicates the specific stain and the white arrowhead the basal layer. The bars represent 50 *μ*m.

HOP expression was analysed in other patients by Northern (Figure 1B) and slot (data not shown) blotting. As expected, the predominant band on Northerns migrates around 1000 bp (compared to the ribosomal RNA markers, Figure 1B). HOP expression was found to be decreased in 18 out of 19 tumours compared to normal samples. Expression of HOP was also decreased in involved lymph nodes (L; Figure 1B, patients 11, 13, 14). The expression levels of HOP were assessed by RT-QPCR in a panel of 33 hypopharyngeal carcinomas, for which there were 23 matched normal tissues (Figure 2

). HOP was expressed at high levels in normal samples (21 out of 23) and strikingly decreased in all tumour samples (average fold change=27). *In situ* hybridisation (ISH) of frozen sections was used to localise HOP mRNA expression (Figure 3

). HOP expression was detected in the suprabasal layer of the epithelium of normal tissue (uvula) (3A, antisense; 3B, haematoxylin/eosin). There was no significant signal with the sense probe and normal tissue, or with the antisense probe and several tumour samples (data not shown). The expression pattern is compatible with a role for HOP in epithelial differentiation and suggested that HOP expression might be associated with the degree of differentiation of the tumours. Homeodomain-only protein expression, measured by RT-QPCR, indicates that there is no statistically significant difference according to the histopathological differentiation status of HNSCC tumours (*P*=0.16; Figure 2).

## DISCUSSION

We have shown that HOP expression is strikingly decreased in hypopharyngeal HNSSC, in 44 out of 46 patients analysed. We also observed decreased HOP expression in tumours by microarray analysis (Cromer *et al*, 2004). HOP was found to be expressed in the suprabasal layer of the epithelium, pointing to a role in keratinocyte differentiation.

There is a striking recent convergence of results from several laboratories that indicate that HOP is an important new tumour suppressor. Several studies showed that HOP (mOB1) is expressed during mouse development (Adu *et al*, 2002) and is involved in cardiac development (Chen *et al*, 2002; Shin *et al*, 2002). More recently, HOP was shown to be a choriocarcinoma suppressor gene involved in cytotrophoblast differentiation (Asanoma *et al*, 2003), and to be underexpressed in primary lung tumours compared with normal lung (Chen *et al*, 2003). Homeodomain-only protein is located in a chromosomal region (4q11–q12) that is frequently deleted in solid tumours, including lung tumours, hepatocellular carcinoma and bladder cancer (Petersen *et al*, 1997; Koo *et al*, 1999; Sakakura *et al*, 1999; Wong *et al*, 1999). Our study provides evidence for a role of HOP in HNSCC.

HOP consists of 73 amino acids with an unusual 60 amino-acid homeodomain, and is the smallest homeodomain protein to date. Homeodomain proteins are important for embryogenesis and development (reviewed in (Chi and Epstein, 2002; Duboule, 1994)). HOP does not possess conserved homeodomain residues needed for DNA binding, but modulates cardiac genes expression by direct interaction with SRF and inhibition of SRF binding to DNA (Chen *et al*, 2002; Shin *et al*, 2002).

HOP is widely expressed in mouse and human tissues, suggesting that it may have an important role in many cell types. Using adult human tissue dot blots (Clontech ref PT3307-1), we found expression in oesophagus, lung, placenta, thyroid gland, foetal lung and brain (in decreasing order, data not shown). The cellular localisation of HOP is predicted to be mainly nuclear. We found, by immunocytochemistry with our specific antibodies and HaCat spontaneously immortalized keratinocytes, that endogenous HOP is nuclear (data not shown), in agreement with others (Chen *et al*, 2002; Shin *et al*, 2002).

HOP expression has been detected in primary cultures of normal lung but not in lung tumour-derived cell lines (Chen *et al*, 2003). Similarly, we detected, by semiquantitative RT–PCR, expression in primary keratinocytes and HaCat cells, but not in a panel of 15 HNSCC-derived cell lines (data not shown). Reintroduction of HOP in choriocarcinoma cell lines inhibits cell proliferation and tumour formation in nude mice (Asanoma *et al*, 2003). We report that HOP expression is decreased in HNSCC tumours compared to their normal matched samples, consistent with studies in other cancer types (Asanoma *et al*, 2003; Chen *et al*, 2003). The reduction of HOP/LAGY expression in lung squamous cell carcinoma is correlated with increasing TNM staging. A complete loss of expression of HOP in two poorly differentiated lung tumour samples has also been reported (Chen *et al*, 2003). In our study, there is no statistically significant association with tumour differentiation, suggesting that loss of HOP expression is important in all tumours despite the degree of differentiation. We have shown that HOP is expressed in the suprabasal layer of the upper aerodigestive tract epithelium, suggesting that it is involved in keratinocyte differentiation. HOP is potentially responsible for the balance between proliferation and differentiation of cardiomyocytes in developing heart (Shin *et al*, 2002). Overexpression Of HOP in choriocarcinoma cell lines induces the expression of CSH1, a marker of differentiated syncytiotrophoblasts (Asanoma *et al*, 2003). Taken together, the results in HNSCC and in other cancer types indicate that HOP could be an important tumour suppressor gene in a wide range of solid tumours.
